# Underestimation of Self-Reported Smoking Prevalence in Korean Adolescents: Evidence from Gold Standard by Combined Method

**DOI:** 10.3390/ijerph15040689

**Published:** 2018-04-05

**Authors:** Jun Hyun Hwang, Jong Yeon Kim, Do Hoon Lee, Hye Gyoun Jung, Soon-Woo Park

**Affiliations:** 1 Department of Preventive Medicine, Catholic University of Daegu School of Medicine, Daegu 42472, Korea; pmdr213@cu.ac.kr (J.H.H.); kom824@cu.ac.kr (J.Y.K.); jhygn@daum.net (H.G.J.); 2 Department of Laboratory Medicine, Center for Diagnostic Oncology, National Cancer Center, Goyang-si 10408, Korea; dhlee@ncc.re.kr

**Keywords:** adolescents, cotinine, self-reported survey, smoking, validity

## Abstract

The objective of this study was to evaluate the validity of self-reported smoking prevalence in Korean adolescents by using an improved gold standard by a combined method. Using a stratified sampling method, we selected 13 schools from among 397 high schools that participated in the 2015 Korean Youth Health Risk Behavior Web-Based Survey (KYRBS). A second survey (repeated self-reporting questionnaire and urinary cotinine test) was conducted on 1058 students who completed the KYRBS. The gold standard of current smoker was defined as those either self-reporting as a smoker in the second survey or having a urinary cotinine concentration ≥50 ng/mL. The current smoking prevalence in the first survey (KYRBS) was 7.9% (boys 16.5% and girls 1.8%), which was lower than the results based on gold standard (11.3% total, boys 21.9% and girls 3.7%). The sensitivity and specificity of self-reported smoking status was 62.5% and 99.0%, respectively. In particular, the sensitivity of girls (43.5%) was lower than that of boys (67.0%). The self-reported smoking prevalence in Korean adolescents was underestimated, particularly among girls. Careful attention should be paid to interpreting adolescents’ smoking prevalence, and supplementary surveys or periodic validity tests need to be considered in Asian countries.

## 1. Introduction

Generally, self-reported questionnaires, which are generally used for computing smoking statistics, provide underestimated outcomes compared to the actual smoking prevalence [1]. This is because smoking is perceived as a socially undesirable behavior; thus, some individuals, particularly women and adolescents, tend to hide their smoking status in self-reported questionnaires [2,3,4,5,6]. However, a systematic review of adults reported that the absolute difference between self-reported smoking prevalence and biochemically measured smoking prevalence ranged from 1–47% depending on the subjects and methods of survey, indicating considerably varying validities of self-reported surveys across studies [1].

The gold standard used by previous studies for assessing the validity of smoking surveys were mostly biochemical tests using urine, saliva, serum, and hair specimens. However, when only a biochemical test is used as the gold standard to assess the validity of self-reported smoking surveys, a misclassification error may arise, where passive smokers are classified as smokers while occasional smokers are classified as nonsmokers [1,7]. Various attempts have been made to address such shortcomings, such as modifying the cutoff value for biochemical tests, assessing recent smoking status (within 3–7 days) considering the half-life of biomarkers, or using various types of specimens [1,8,9]. Nevertheless, only using biochemical tests as a gold standard cannot be a fundamental solution to resolve misclassification errors because sensitivity and specificity, the key indices for assessing validity, are in a trade-off relationship. In particular, a misclassification error, where smokers are classified as nonsmokers, may be more frequent among adolescents than in adults, as younger individuals are more likely to be occasional smokers [10].

School-based self-reported smoking surveys have employed the bogus pipeline method as a psychological approach to curb social desirability biases [11,12,13,14]. This method induces honest responses from the respondents by notifying them beforehand that their responses will be crosschecked with biochemical tests. One benefit of this method is that it can detect even those occasional smokers who cannot be confirmed via biochemical tests due to infrequent smoking that exceeds the half-life of biomarkers or having low levels of biomarkers that are below the cutoff values. However, there is still a possibility that results obtained through the bogus pipeline method are not true. Thus, employing results from both biochemical tests and the bogus pipeline method as a gold standard may minimize misclassification errors as compared to when either method is used alone. To our knowledge, no study has employed this combined method.

Therefore, we attempted to assess the validity of anonymous self-reported smoking surveys by applying a new gold standard utilizing the combined method (repeated self-reporting questionnaire and urinary cotinine test) on some respondents to the Korean Youth Health Risk Behavior Web-Based Survey (KYRBS), Korea’s nationally representative survey. Additionally, we attempted to identify characteristics of smoking adolescents who provide false responses in smoking surveys.

## 2. Materials and Methods

### 2.1. Study Design and Population

We used a subgroup of participants in the 11th Korea Youth Risk Behavior Web-Based Survey (KYRBS-XI) conducted in 2015 by the Korea Centers for Disease Control and Prevention. The KYRBS uses a stratified multistage probability sampling design to produce nationally representative statistics on health behaviors in Korean adolescents. A total of 68,043 students from 797 schools (400 middle schools and 397 high schools) completed the KYRBS-XI (response rate = 96.7%) [15].

Among the high schools participating in KYRBS-XI, we selected 13 high schools using a stratified sampling method considering schools’ location (seven metropolitan city schools and six city schools), characteristics (11 general and two vocational schools), and sex (six coeducational schools, two boys schools, five girls schools). Of the 1214 students in the 13 high schools participating in the KYRBS-XI, 12th grade students (79 students) from two schools that refused to participate in the repeated survey due to academic constraints were excluded. Of the 1135 students in targeted populations, 1111 students participated in a repeated survey, and the final study population who completed the urinary cotinine test was 1059 students (participation rate 93.3%) after excluding students who refused the urinary cotinine test (52 students).

The KYRBS-XI (hereafter first survey) and second survey were conducted in June 2016, and June or July 2016, respectively, and the median time between the two surveys was 15 days (interquartile range 12–28 days). There was no specific school schedule including school trips or vacations that influenced students’ smoking habits. The first survey was carried out in the same way as the usual KYRBS-XI (web-based anonymous self-reported questionnaires under supervision of school staff). Those who participated in the first survey were not informed about the second survey for validation of self-reported smoking status until after they completed the first survey. Upon completing the first survey, they were told that resurvey for validation of self-reported smoking status would proceed; they were advised to turn in an enclosed identification number to the investigator for matching individuals between surveys. The second survey was composed of an anonymous self-reported questionnaire and a urinary cotinine test. Before the second survey, the participants were told about the urinary cotinine test for their smoking validation by well-trained surveyors, and they assured the students of the anonymity and confidentiality of their survey results. The results of the first and second survey were matched individually by identification number. This study design was approved by the Institutional Review Board of the Daegu Catholic University Medical Center (CR-15-049).

### 2.2. Measures

In both the first and second survey, a self-reported current smoker was defined as a person who had smoked one or more days in the most recent 30 days using the following question: “During the past 30 days, on how many days did you smoke cigarettes?”

During the second survey, midstream urinary samples among students who completed the questionnaire were collected. The samples were analyzed by a liquid chromatography–tandem mass spectrometry (LC-MS-MS) system on an ABSciex API 4000 using the TurboIonSpray interface and multiple reaction monitoring (Applied Biosystems, Foster City, CA, USA) [16]. Students with urinary cotinine concentrations of ≥50 ng/mL, the level recommended by the Society for Research on Nicotine and Tobacco, were considered cotinine-verified smokers [17].

In addition to this individual classification of current smokers, estimated smoking prevalence was suggested as a subsidiary method to assure the confidentiality and anonymity using the representative value rather than the individual response to smoking questionnaire questions. Estimated smoking prevalence based on the mode value of the number of smoking classmates was calculated by asking the following question: “How many students smoke in your classroom?” In cases where the mode value was 0 or extreme (ex. 13 in 10th grade girls), we selected the second most frequent value. When there were two mode values in a class, we applied the mean of the two mode values. Estimated smoking prevalence was calculated by dividing the sum of the estimated number of smoking classmates by the total number of the students in the selected class (*N* = 1163). Since sex-specific smoking prevalence in co-educational classes cannot be calculated due to not asking the number of smokers by sex in each class, students in co-educational classes (six classes in two schools) were excluded from the calculation to estimate sex-specific smoking prevalence.

Confidentiality of survey environment and friends’ cigarette smoking were considered as environmental factors affecting underreporting of smoking. Confidentiality of survey response was assessed using responses to the following question: “Do you think that the first survey (KYRBS-XI) results could leak out to teachers or parents?” Closest friends’ cigarette smoking was assessed using responses to the following question: “Do any of your closest friends smoke tobacco?” Participants were provided with three possible answers: (1) none of them; (2) some of them; and (3) most/all of them.

### 2.3. Statistical Analysis

Current smokers were assessed using the following four methods: (1) self-reported in first survey; (2) self-reported in second survey; (3) urinary cotinine concentration ≥50 ng/mL in second survey; (4) combined method in second survey (either by self-reported questionnaires and/or urinary cotinine concentration). The combined method was used as a gold standard for the classification of current smokers.

Validation of anonymous self-reported smoking status was evaluated in two ways using three indicators (sensitivity, specificity, Kappa value). First, results between self-reported questionnaires and urinary cotinine concentration within the second survey were compared. Since students knew about the accompanying biochemical test before filling out the questionnaires, this method evaluated the validity of the questionnaire survey conducted by the bogus pipeline method. Second, the results of self-reported questionnaires in the first survey were compared with those of the second survey with three different criteria: (1) self-reported questionnaires; (2) urinary cotinine concentration; and (3) the combined method (gold standard). This comparison was newly designed to assess the validity of anonymous self-reported smoking status by applying improved criteria.

Among current smokers based on gold standard criteria (three students were excluded due to missing data from question item about the confidentiality of the survey environment), multivariate logistic regression was performed to investigate the characteristics of respondents providing false information. Sex, grade, confidentiality of survey environment, friends’ cigarette smoking, and cotinine level were included in statistical model; cotinine levels were classified as tertiles.

For comparison with the validity of anonymous self-reported smoking status, the validity of both sensitive items (self-reported current drinking status) and non-sensitive items (self-reported height) were also evaluated between the first and second surveys. The validity of self-reported drinking status was evaluated in the same way as the smoking status, using three indicators (sensitivity, specificity, Kappa value). Paired *t*-test for equivalence (equivalence bounds: 1 cm) was used to evaluate validity of self-reported height. All analyses were performed using SPSS version 19.0 (IBM, Armonk, NY, USA), and a *p*-value of <0.05 was considered significant.

## 3. Results

The proportion of higher grade participants was significantly higher in girls (12th grade: 41.3% for girls vs. 16.2% for boys; *p* < 0.001). The proportion of smoking closest friends in girls was significantly lower than that in boys (*p* < 0.001). There was no statistically significant difference in perceived threats to the confidentiality of the survey response between boys and girls (see Supplementary Materials Table S1).

The prevalence of current cigarette smoking in the first survey using a self-reported questionnaire was 7.9% (boys 16.4%, girls 1.8%), which was lower than those of 9.0–11.3% in the second survey using the self-reported questionnaire, urinary cotinine concentration of ≥50 ng/mL, or the estimated prevalence based on the mode value of the number of smoking classmates. Of all of the methods, the overall prevalence of current cigarette smoking in the study population was the highest when smoking status was assessed using the combined method (either self-reported questionnaire and/or urinary cotinine test, gold standard) in the second survey; 3.4 percentage point (boys 5.5 percentage point, girls 1.9 percentage point) higher than the results from the first survey (Table 1).

Table 2 and Table 3 show the validity of self-reported current smoking status based on sensitivity, specificity, and Kappa values. In the second survey, the sensitivity of self-reported smoking based on urinary cotinine concentration (cutoff value: 50 ng/mL) was 91.6% (95% confidence interval: 84.1, 96.3), the specificity was 97.4% (95% confidence interval: 96.2, 98.3), and the Kappa value was 0.823 (95% confidence interval: 0.765, 0.882). Although there was no statistical significant difference between sexes, the sensitivity and Kappa value in girls were lower than those in boys (Table 2).

When the validity of the self-reported current smoking status in the first survey was evaluated through comparison with the second survey results with three criteria. The sensitivity based on the combined method was the lowest at 62.5% (95% confidence interval: 53.2, 71.2), and there was a difference of about 5% as compared with other methods. The results of stratification by sex also showed the lowest sensitivity in the combined method. According to this combined method, 33.0% of boy smokers and 56.5% of girl smokers reported that they did not smoke cigarettes in the first survey. Although there was no statistical significant difference between sexes, sensitivity in girls was lower than that in boys by 23.5 percentage points. When sensitivity was evaluated by a single method, the difference in sensitivity between sexes was also about 20 percentage points. Overall, the specificity of self-reported smoking was more than 95.0% regardless of sex or evaluation method (Table 3).

Compared with the second self-reported questionnaire, the sensitivity for self-reported current drinking status in the first survey was similar to that of smoking (drinking 69.9% vs. smoking 67.7%), but the specificity of the alcohol questionnaire was lower than that of smoking (drinking 91.0% vs. smoking 99.1%). As in the result of self-reported smoking status, the sensitivity for self-reported drinking status in girls was lower than that in boys. However, the sex difference in sensitivity was larger in the questionnaire for questions about smoking than for questions about alcohol (smoking 17.3 percentage points vs drinking 7.2 percentage points) (see Supplementary Materials Table S2). Unlike sensitive items such as smoking or alcohol, there was no difference in self-reported height between the first and second surveys (see Supplementary Materials Table S3).

Of 120 current smokers based on the combined method, girls were 2.64 times more likely to hide their smoking status in self-reported surveys than boys. However, this significant result became non-significant after multivariate analysis. There was no statistically significant association between hiding smoking status and perceived threat to the confidentiality of the survey responses. The risk of smokers hiding their smoking status significantly increased from 1.58 (95% confidence interval: 0.53, 4.71) to 58.21 (95% confidence interval: 6.37, 531.76) when the number of closest friends who smoked decreased. Students with low urinary cotinine concentrations were significantly more likely to hide their smoking status (Table 4).

## 4. Discussion

This study assessed the validity of self-reported smoking surveys in adolescents against a gold standard based on a combined method (repeated survey and urinary cotinine test). About 37.5% of smoking adolescents lied about their smoking status, and the proportion of false responses was higher for girls (56.5%) than for boys (33.0%). In contrast, the specificity of the survey was 99%, suggesting that nonsmokers rarely claimed to be smokers. Although the validity of surveys varies across studies, adolescents’ self-reported smoking prevalence was underestimated compared to the actual smoking rate [2,8,13,18,19,20]. The sensitivity of the smoking survey was 81.6% for ages 12–19 years and 92.0% for ages 20–79 years in the Canadian Health Measures Survey (CHMS) and 60.1% for ages 12–17 years and 89.4% for ages 18–25 years in the US National Household Survey on Drug Abuse (NHSDA), showing that self-reported smoking prevalence is underestimated in greater levels among adolescents compared to adults [2,4]. However, although there were no sex-specific differences in validity in Western countries such as the US and Canada [2,18], large sex differences were evident in Asian countries, such as China, Korea, and Iran, where only about 10% of Korean adult men lied about their smoking status, while more than half of Korean adult women hid their smoking status [5,19,21]. This may be attributable to the fact that sociocultural backgrounds in Asian countries, such as Muslim culture or Confucianism, tend to convey negative perceptions about female smoking [5,22,23,24]. The fact that sex differences in adolescent smoking rates in the Eastern Mediterranean Region, Southeast Asia Region, and Western Pacific Region are higher compared to those in the European Region and Americas Region also supports the presence of a sex difference in the social norm for smoking [22].

In previous studies assessing the validity using a biochemical test alone as a gold standard on Korean adolescents, the sensitivity of the survey was high at about 90%, confirming the validity of self-reported smoking surveys [25,26]. However, when we applied the combined method as a gold standard, the sensitivity of the self-reported smoking survey dropped by about 5 percentage points as compared to when only the biochemical test was used as a gold standard, while the specificity did not vary significantly. In other words, the sensitivity of previous studies that only used a biochemical test as a gold standard was overestimated, while the difference between the actual smoking rate and self-reported smoking prevalence was underestimated.

In the present study, adolescents whose friends do not smoke or who had low urinary cotinine concentrations were more likely to lie about their smoking status. Because smoking is perceived as a socially undesirable behavior, occasional smokers who have only recently begun to smoke or those who do not have smoking friends are thought to have attempted to hide their smoking status from others and thus lied about it in the surveys. The NHSDA in the US also showed that adolescents who had more smoking friends were less likely to give false responses about their smoking status [4]. Social perception may affect behaviors, and the degree of false responses may differ according to the degree of social acceptance. In our study, students also tended to lie about drinking status, which is another socially undesirable behavior, and girls tended to lie about it more often. However, when we compared the first and second self-reported surveys, the sex-specific difference in sensitivity was smaller in drinking items than in smoking items (smoking 17.3 percentage points vs. drinking 7.2 percentage points). This is speculated to reflect the fact that the social mood is more lenient with drinking than smoking, as evidenced by a higher drinking rate than smoking rate in Korean adolescents and a smaller gap in drinking prevalence between sexes [15]. In contrast, there were no differences in the first and second survey results in socially insensitive items, such as height.

This study has a few limitations. First, the second survey was conducted about 15 days (median) after the first survey, and the students’ smoking status may have changed within that period. However, we administered the survey during the semester to avoid special circumstances that may affect students’ smoking status, such as school trips or summer vacation. We performed the surveys before the summer break in Korea, considering that smoking rate among adolescents tends to rise during school breaks when adult monitoring is difficult [27]. The National Health and Nutrition Examination Survey, Health Survey for England, and CHMS also administered the questionnaire and biochemical test to assess the validity of self-reported smoking survey in about 2-week, 1-week, and average 13-day intervals, respectively [2,28].

Second, it may be difficult to generalize the findings of our study because we did not include all of the participants of the KYRBS-XI, a nationally representative survey of Korean adolescents, in our investigation. Because it is practically difficult to investigate all the KYRBS-XI respondents (about 70,000), we selected the schools via a stratified sampling method considering the size of the school’s location, characteristics, and co-educational status to ensure the representativeness of the sample. Nonetheless, the results of this study cannot be generalized to non-Korean adolescents. However, our study can provide meaningful data to Asian countries because, to the best of our knowledge, there has been no validity study using a biochemical test on more than 1000 adolescents in an Asian country.

There have been differences in validity results across countries (Valid: USA, Canada, Mexico; Not valid: Poland, Brazil, Korea, Iran) [2,5,8,20,21,28]. Asian countries display negative perceptions about female and adolescent smoking primarily due to their sociocultural backgrounds, such as Muslim culture and Confucianism. Additionally, the widespread denormalization of smoking caused by tobacco control policies may affect the validity of self-reported smoking surveys. In Korea, adolescent smoking rates have dropped by about 50% within the past 10 years (12.8% in 2006, 6.3% in 2016), with a greater drop among girls [29]. The number of students who do not have a close friend who smokes increased from 53.4% in 2014 to 60.1% in 2016 [29]. In light of our finding that students who do not have a close friend who smokes are more likely to give false responses, the interaction between a growing negative perception about smoking and a decline in the number of smoking friends may have led to a rise of false reports in self-reported questionnaires, thereby elevating the degree of underestimation of smoking prevalence.

In Asian countries, investigators should reconsider conducting their validity studies during periods of rapid decline of smoking prevalence or periods of changes in social norms concerning smoking. Administering biochemical tests may be practically challenging due to high costs or ethical reasons, so it may be useful to regularly perform surveys to estimate smoking prevalence using additional methods. In China, the adolescent smoking rate has been estimated using the capture-recapture method based on an additional survey that asked parents about their child’s smoking habits [19]. In our study, the smoking prevalence estimated by investigating the number of smoking students was closer to the actual smoking rate than was the self-reported smoking prevalence, suggesting that methods other than biochemical tests can be used.

## 5. Conclusions

Self-reported smoking prevalence in adolescents considerably underestimates the actual smoking prevalence in adolescents, where about one-third of boys and one-half of girls who smoke lie about their smoking status in smoking surveys. Thus, investigators should be careful when interpreting adolescent smoking prevalence, and supplementary surveys or regular validity tests should be performed to compensate for the limitations of self-reported smoking surveys.

## Figures and Tables

**Table 1:** Current cigarette smoking prevalence according to the survey method.

Survey	Method	Total (*N* = 1059)		Boys (*N* = 444)		Girls (*N* = 615)	
		*N*	%	*N*	%	*N*	%
First survey	Self-reported questionnaire	84	7.9	73	16.4	11	1.8
Second survey	Self-reported questionnaire (A)	112	10.6	93	21.0	19	3.1
	Urine cotinine (B) ^a^	95	9.0	79	17.8	16	2.6
	Combined method (A and/or B, Gold standard)	120	11.3	97	21.9	23	3.7
	Estimated prevalence ^b^	115	9.9	75.5 ^c^	20.0	27 ^c^	4.6

^a^ Cutoff value of urinary cotinine concentration: 50 ng/mL. ^b^ Estimated prevalence from the mode by asking following question: “How many students smoke in your classroom?” In case of a mode value that was 0 or extreme (ex. 13 in 10th grade girls), we selected the second most frequent value. In a case of two mode values, we applied the mean of the two mode values. Estimated smoking prevalence was calculated by dividing the sum of the estimated number of smoking classmates by the total number of the students in selected class (*N* = 1163). ^c^ Co-educational classes (*N* = 204) were excluded in calculation of the number and percentage of the estimated smokers. The number of the denominator in boys and girls was 378 and 581, respectively.

**Table 2:** Validity evaluation of self-reported current cigarette smoking prevalence within the second survey.

	Second Survey		Self-Reported Questionnaire					
			Total (*N* = 1059)		Boys (*N* = 444)		Girls (*N* = 615)	
Second Survey			Current Smoker *N* (%)		Current Smoker *N* (%)		Current Smoker *N* (%)	
			Yes	No	Yes	No	Yes	No
Urine cotinine ^a^	Current smoker	Yes	87 (91.6)	8 (8.4)	75 (94.9)	4 (5.1)	12 (75.0)	4 (25.0)
		No	25 (2.6)	939 (97.4)	18 (4.9)	347 (95.1)	7 (1.2)	592 (98.8)
	Validity indicator		Value	95% CI	Value	95% CI	Value	95% CI
	Sensitivity (%)		91.6	84.1, 96.3	94.9	87.5, 98.6	75.0	47.6, 92.7
	Specificity (%)		97.4	96.2, 98.3	95.1	92.3, 97.1	98.8	97.6, 99.5
	Kappa		0.823	0.765, 0.882	0.842	0.778, 0.906	0.677	0.497, 0.857

Abbreviation: CI, confidence interval. ^a^ Cutoff value of urinary cotinine concentration: 50 ng/mL.

**Table 3:** Validity evaluation of self-reported current cigarette smoking prevalence between the first and second surveys.

	First Survey		Self-Reported Questionnaire					
			Total (*N* = 1059)		Boys (*N* = 444)		Girls (*N* = 615)	
Second Survey			Current Smoker *N* (%)		Current Smoker *N* (%)		Current Smoker *N* (%)	
			Yes	No	Yes	No	Yes	No
Self-reported questionnaire (A)	Current smoker	Yes	75 (67.0)	37 (33.0)	65 (69.9)	28 (30.1)	10 (52.6)	9 (47.4)
		No	9 (1.0)	938 (99.1)	8 (2.3)	343 (97.7)	1 (0.2)	595 (99.8)
	Validity indicator		Value	95% CI	Value	95% CI	Value	95% CI
	Sensitivity (%)		67.0	57.4, 75.6	69.9	59.5, 79.0	52.6	28.9, 75.6
	Specificity (%)		99.1	98.2, 99.6	97.7	95.6–99.0	99.8	99.1, 100.0
	Kappa		0.742	0.671, 0.813	0.734	0.653, 0.816	0.659	0.462, 0.856
Urine cotinine (B) ^a^	Current smoker	Yes	64 (67.4)	31 (32.6)	57 (72.2)	22 (27.9)	7 (43.8)	9 (56.3)
		No	20 (2.1)	944 (97.9)	16 (4.4)	349 (95.6)	4 (0.7)	595 (99.3)
	Validity indicator		Value	95% CI	Value	95% CI	Value	95% CI
	Sensitivity (%)		67.4	57.0, 76.6	72.2	60.9, 81.7	43.8	19.8, 70.1
	Specificity (%)		97.9	96.8, 98.7	95.6	93.0, 97.5	99.3	98.3, 99.8
	Kappa		0.689	0.609, 0.769	0.699	0.609, 0.788	0.508	0.276, 0.741
A and/or B (Gold standard)	Current smoker	Yes	75 (62.5)	45 (37.5)	65 (67.0)	32 (33.0)	10 (43.5)	13 (56.5)
		No	9 (1.0)	930 (99.0)	8 (2.3)	339 (97.7)	1 (0.2)	591 (99.8)
	Validity indicator		Value	95% CI	Value	95% CI	Value	95% CI
	Sensitivity (%)		62.5	53.2, 71.2	67.0	56.7, 76.2	43.5	23.2, 65.5
	Specificity (%)		99.0	98.2, 99.6	97.7	95.5, 99.0	99.8	99.1, 100.0
	Kappa		0.708	0.635, 0.781	0.710	0.627, 0.794	0.578	0.379, 0.777

Abbreviation: CI, confidence interval. ^a^ Cutoff value of urinary cotinine concentration: 50 ng/mL.

**Table 4:** Prevalence and adjusted odds ratios of false-negative reporters about smoking status according to characteristics in current smokers ^a^.

Characteristic		False-Negative Reporters		Crude OR	95% CI	Adjusted OR ^b^	95% CI
		Cases/Subjects	Prevalence (%)				
Sex	Boys	32/97	33.0	Referent		Referent	
	Girls	13/23	56.5	2.64	1.05, 6.67	2.27	0.70, 7.40
Grade	12th	17/33	43.6	Referent		Referent	
	11th	9/47	19.2	0.31	0.12, 0.80	0.44	0.13, 1.56
	10th	19/34	55.9	1.64	0.65, 4.14	1.66	0.49, 5.60
Perceived threat to confidentiality	No	19/58	32.8	Referent		Referent	
	Yes	26/62	41.9	1.48	0.70, 3.12	1.48	0.56, 3.94
Closest friend smoking	Most/All	11/59	18.6	Referent		Referent	
	Some	18/44	40.9	3.02	1.24, 7.35	1.58	0.53–4.71
	No	16/17	94.1	69.80	8.35, 583.661	58.21	6.37, 531.76
Urine cotinine ^c^	3rd tertile	11/40	27.5	Referent		Referent	
	2nd tertile	10/40	25.0	0.88	0.32, 2.38	1.12	0.31, 4.02
	1st tertile	24/40	60.0	3.96	1.55, 10.11	3.96	1.11, 14.13

Abbreviation: CI, confidence interval; OR, odds ratio. ^a^ Current smokers based on the combined method (either self-reported questionnaire and/or urinary cotinine test, gold standard). ^b^ Adjusted for all covariates. ^c^ Cutoff points of first tertile and second tertile were 131.1 ng/mL and 695.0 ng/mL.

## Supplementary Materials

The following are available online at http://www.mdpi.com/1660-4601/15/4/689/s1, Table S1: General Characteristics of Study Population by Sex, Table S2: Validity Evaluation of Anonymous Self-reported Current Drinking Between First and Second Survey, Table S3: Validity evaluation of Anonymous Self-reported Height Between First and Second Survey.
