# Supplementary material for: Underestimation of Self-Reported Smoking Prevalence in Korean Adolescents: Evidence from Gold Standard by Combined Method
Source: Int J Environ Res Public Health. 2018 Apr 5;15(4):689. doi: 10.3390/ijerph15040689 (PMC5923731; doi:10.3390/ijerph15040689)
Supplement: Supplementary file 1 [file ijerph-15-00689-s001.pdf]

Supplementary Materials

Table S1. General Characteristics of Study Population by Sex

		Boys (N=444)		Girls (N=615)		*P* value
		N	%	N	%	
Grade	10th	186	41.9	166	27.0	<0.001
	11th	186	41.9	195	31.7	
	12th	72	16.2	254	41.3	
Perceived threat to confidentiality [a]	Yes	104	23.5	132	21.5	0.455
	No	339	76.5	481	78.5	
Closest friend smoking	None	122	27.5	462	75.1	<0.001
	Some	242	54.5	139	22.6	
	Most/All	80	18.0	14	2.3	

[a] 1 boy and 2 girls were excluded due to missing data of question item

Table S2. Validity Evaluation of Anonymous Self-reported Current Drinking Between First and Second Survey

2nd Survey \ 1st Survey		Total (N=1,059) Current drinker N (%)		Boys (N=444) Current drinker N (%)		Girls (N=615) Current drinker N (%)	
		Yes	No	Yes	No	Yes	No
Current drinker	Yes	183(69.9)	79(30.2)	99(73.3)	36(26.7)	84(66.1)	43(33.9)
	No	72(9.0)	725(91.0)	29(9.4)	280(90.6)	43(8.8)	445(91.2)
Validity indicator		Value	95% CI	Value	95% CI	Value	95% CI
Sensitivity (%)		69.9	63.9, 75.3	73.3	65.0, 80.6	66.1	57.2, 74.3
Specificity (%)		91.0	88.8, 92.9	90.6	86.8, 93.6	91.2	88.3, 93.6
Kappa		0.614	0.558, 0.669	0.649	0.571, 0.727	0.573	0.493, 0.654

Abbreviation: CI, confidence interval

Table S3. Validity evaluation of Anonymous Self-reported Height Between First and Second Survey

Sex	N[a]	1st Survey mean (SD)	2nd Survey mean (SD)	Difference mean (SD)	Equivalence test [b]
Boys	434	173.7 (6.1)	173.9 (5.9)	-0.2 (1.6)	Equivalent
Girls	601	160.6 (5.2)	160.7 (5.4)	-0.1 (1.6)	Equivalent

[a] 10 boys and 14 girls were excluded due to missing data of height

[b] Paired T-test for equivalence (equivalence bounds: ±1 cm)
